# Market efficiency of cryptocurrency: evidence from the Bitcoin market

**DOI:** 10.1038/s41598-023-31618-4

**Published:** 2023-03-23

**Authors:** Eojin Yi, Biao Yang, Minhyuk Jeong, Sungbin Sohn, Kwangwon Ahn

**Affiliations:** 1 Seoul Business School, aSSIST University, Seoul, Republic of Korea; 2 Antai College of Economics and Management, Shanghai Jiao Tong University, Shanghai, China; 3 Department of Industrial Engineering, Yonsei University, Seoul, Republic of Korea; 4 Center for Finance and Technology, Yonsei University, Seoul, Republic of Korea; 5 Department of Economics, Sogang University, Seoul, Republic of Korea

**Keywords:** Nonlinear phenomena, Quantum information, Statistics

## Abstract

This study examines whether the Bitcoin market satisfies the (*weak-form*) efficient market hypothesis using a quantum harmonic oscillator, which provides the state-specific probability density functions that capture the superimposed Gaussian and non-Gaussian states of the log return distribution. Contrasting the mixed evidence from a variance ratio test, the high probability allocated to the ground state suggests a near-efficient Bitcoin market. Findings imply that as Bitcoin evolves into an efficient market, speculators might encounter difficulty in exploiting profitable trading strategies. Furthermore, when policymakers initiate tight regulations to control the market, they should closely monitor market efficiency as an index of price distortion.

## Introduction

“The rapidly evolving world of digital money”

*New York Times, March 18, 2015.*

The rapidly growing market capitalization and extreme price fluctuations of the cryptocurrency market have prompted policymakers and economists to define cryptocurrency within a financial and economic context. Many studies use Bitcoin, which has the longest history and the largest market capitalization, as a benchmark for cryptocurrency research. Considering the potential impact of transitioning to a paperless *digital society* on the financial market and real economy, it is important to analyze the Bitcoin market with quantitative evidence.

One of the hottest debates in finance and economics is whether the market is efficient. Over time, numerous studies have investigated the market efficiency of various assets for asset pricing, risk management, and asset allocation. Since Fama^1^, this landmark issue has been dealt with based on the concept of market information and return predictability. According to the efficient market hypothesis (EMH), since investors’ rational expectations based on relevant information are quickly reflected in market prices, price fluctuations are unpredictable. This has been tested in stock, bond, foreign exchange, and other emerging markets. The market information is divided into three levels: (1) historical price and return, (2) all publicly available information (in the public domain), and (3) information privately known or known only to a limited group of market participants. The weak-form EMH states that price is unpredictable using the first level of information.

We extend the literature by examining the weak-form EMH of Bitcoin. Some recent studies reported that the Bitcoin market does not follow EMH^2–10^. However, other studies documented that the Bitcoin market follows EMH^11–14^. For example, in opposition to Urquhart^10^’s findings, Nadarajah and Chu^15^ found that a power transformation of Bitcoin returns is weakly efficient. Moreover, several studies have reported that the Bitcoin market is still transitional as it is currently inefficient; but steadily improving^16–21^. For instance, Sigaki et al.^22^ provided evidence of the changes in informational efficiency of the cryptocurrency market, which could have originated from collective phenomena in marketplaces^23^. Essentially, there is still mixed evidence on the efficiency of the Bitcoin market.

Among the various methods for measuring market efficiency, the variance ratio (VR) test is a well-known standard based on the random walk hypothesis^24^; the increments of log price series are Gaussian white noise^25^. We first examine whether the VR test is suitable for testing the EMH for the Bitcoin market. Then, we propose an alternative analytical framework based on quantum mechanics, i.e., the quantum harmonic oscillator (QHO). This modeling framework considers the market forces affecting price changes from short-term fluctuations to long-term equilibrium^26^. In particular, the solution of the QHO model is a superposition of infinite eigenstates, which form an orthogonal basis encompassing all distribution functions. As a result, our model nests the random walk as a ground-state solution. Accordingly, we analyze the market efficiency of Bitcoin by estimating the probability allocated to the ground state of the QHO model, i.e., the Gaussian distribution.

When share prices fully reflect the information contained in historical prices, consistent alpha generation is impossible. So, does the Bitcoin market work efficiently? Put differently, does the log of Bitcoin prices follow a random walk? This study provides novel evidence to the extant debate on the efficiency of the Bitcoin market: (1) “What are the limitations of the VR test to examine the EMH?”; (2) “Can QHO overcome the limitations of the VR test?”; and (3) “How to economically interpret market efficiency evaluated through quantum mechanics?” In a nutshell, we provide evidence supporting the efficient market characteristics of Bitcoin using annual fluctuations of the ground state probability, considering the Bitcoin price regimes. We also explain the rationale behind our findings using market measures such as the liquidity index. Finally, we provide implications for both investors and policymakers regarding the difficulty in exploiting profitable trading strategies and an index of the price distortion, respectively.

This paper is organized as follows: Section "Data and methodology" describes the data and methodology, Section "Results and discussion" discusses the results, and Section "Conclusions" concludes the paper.

## Data and methodology

### Data

The daily price of Bitcoin is collected from Quandl.com. The data of the other assets, for comparison purposes, are taken from the World Gold Council (gold) and Federal Reserve Bank of St. Louis (USD/EUR exchange rate and S&P 500 index). The period of data collection was from September 01, 2010, to March 31, 2019 (3,134 days). To match the trading dates of Bitcoin and the other assets, we obtained a total of 2,134 observations of daily data (While the Bitcoin market is constantly open (24 hours every day), data for the other comparative assets were available only on trading days.). Then, we took the first difference in log prices and annualized the return as

$${x}_{t}=252.5 \mathrm{ln}\left(\frac{{p}_{t}}{{p}_{t-1}}\right),$$

where ${x}_{t}$ and ${p}_{t}$ are the annualized log return and price at day $t$, respectively.

Table 1 shows that the mean and standard deviation of Bitcoin’s log returns are quite large, compared to the other assets. The Bitcoin returns show positive skewness, implying investors’ risk-loving attitudes. Moreover, the distribution is extremely highly leptokurtic given that excess kurtosis is over 40, indicating that the data do not follow a normal distribution^27–30^.

**Table 1:** Summary statistics of the annualized log returns.

	Obs	Mean	Std	Skewness	Excess kurtosis
Bitcoin	2,133	1.316	21.559	0.925	42.521
Gold		0.005	2.543	− 0.576	7.173
S&P 500		0.114	2.299	− 0.582	4.853
USD/EUR		− 0.016	1.413	− 0.008	1.761

### VR test

The VR test examines whether the log price series follows a random walk^24,31,32^. The key idea is that the variance of the increments in a random walk grows proportionally with the sampling interval $q$. If a time series follows a random walk process, the variance of its $q$ th difference should be $q$ times the variance of its first difference. Accordingly, the variance ratio $VR(q)$ is

$$VR\left(q\right)=\frac{{{\widehat{\sigma }}^{2}\left({Y}_{t}-{Y}_{t-q}\right)}/{q}}{{\widehat{\sigma }}^{2}\left({Y}_{t}-{Y}_{t-1}\right)}$$

with

$$Y_{t} = \mu + Y_{t - 1} + \varepsilon_{t}{\text{ for }}\varepsilon_{t} { }\sim { }i.i.d. N\left( {0,{ }\sigma^{2} } \right)$$

where $Y_{t}$, $\mu$, and $\varepsilon_{t}$ are the log price, drift parameter, and error term. ${\widehat{\sigma }}^{2}$ is the maximum likelihood estimator of variance.

Under the homoscedasticity assumption, the null hypothesis of the VR test is that the log price series follows a random walk: equivalently, $VR\left(q\right)=1$. If the variance ratio is too high $VR\left(q\right)>1$ or too low $VR\left(q\right)<1$, then the log return has either positive or negative autocorrelation.

### QHO

A stochastic differential equation is widely used to describe various random behaviors in the financial market such as mean reversion^33^, stochastic volatility^34^, jump process^35–38^, controlled growth process^39^, and process evolving according to a size-independent proportional growth rate after an exponentially distributed period^40^. Here, we specifically implemented the method introduced by Ahn et al.^26^ and modeled the evolution of the log return distribution. We started with the following stochastic differential equation:

1 $$dx=\mu \left(x,t\right)dt+\sigma \left(x,t\right)d{W}_{t},$$

where $x$ represents an asset return, $\mu (x,t)$ denotes a drift, $\sigma (x,t)$ represents volatility, and ${W}_{t}$ is a standard Wiener process.

The Fokker–Planck (FP) equation is obtained from Eq. (1) by introducing the probability density function (PDF) $\rho (x,t)$ of the random variable $x$ at time $t$:

2 $$\frac{\partial }{\partial t}\rho \left(x,t\right)=\frac{{\partial }^{2}}{\partial {x}^{2}}\left[D\left(x,t\right)\rho \left(x,t\right)\right]+\frac{\partial }{\partial x}\left[\rho \left(x,t\right)\frac{\partial V(x,t)}{\partial x}\right],$$

where $D\left(x,t\right)\equiv {\sigma }^{2}(x,t)/2$ is the diffusion coefficient and $V(x,t)$ is the external potential determining the drift term according to $\mu \left(x,t\right)\equiv \partial V(x,t)/\partial x$. For constant $D$ and time-independent potential $V(x)$, Eq. (2) can be expressed as the FP operator:

3 $$\frac{\partial }{\partial t}\rho \left(x,t\right)=\widehat{L}\rho (x,t),$$

where $$\widehat{L}={V}_{xx}+{V}_{x}\partial /\partial x+D{\partial }^{2}/\partial {x}^{2}$$.

To solve Eq. (3), we examine the steady-state solution ${\rho }_{s}\left(x\right)$ satisfying $\widehat{L}{\rho }_{s}\left(x\right)=0$^41,42^ and introduce the wave function $\Psi \left(x, t\right)$ with Hermitian operator $\widehat{H}$ as

$$\Psi \left(x, t\right)\equiv \frac{\rho \left(x,t\right)}{\sqrt{{\rho }_{s}\left(x\right)}}.$$

Then the FP operator in Eq. (3) leads to

$$\widehat{L}\rho \left(x,t\right)=-\sqrt{{\rho }_{s}\left(x\right)}\widehat{H}\Psi \left(x, t\right)$$

which yields $\widehat{H}=-{V}_{xx}/2+{V}_{x}^{2}/4D-D{\partial }^{2}/\partial {x}^{2}$ and Eq. (3) can be rearranged into the Schrödinger equation with imaginary time $\tau \equiv -i\hslash t$ and mass $m\equiv {\hslash }^{2}/2D$

4 $$i\mathrm{\hslash }\frac{\partial }{\partial \tau }\Psi \left(x, \tau \right)= \widehat{H}\Psi \left(x, \tau \right)\equiv -\frac{{\hslash }^{2}}{2m}\frac{{\partial }^{2}}{\partial {x}^{2}}\Psi (x,\tau )+U\left(x\right)\Psi (x,\tau ),$$

where the effective potential $U\left(x\right)$ is given by^26^

$$U\left(x\right)=U\left(0\right)+\frac{1}{2}k{x}^{2}$$

with $k\equiv {d}^{2}U/d{x}^{2}{|}_{0}$. Near equilibrium, $U\left(x\right),$ is approximated by a harmonic potential and the system reduces to a harmonic oscillator.

Accordingly, the general solution of Eq. (4) bears that of the FP equation and, in particular, the $n$th eigenfunction of the harmonic oscillator is

$${\phi }_{n}\left(x\right)=\frac{1}{\sqrt{{2}^{n}n!}}{\left(\frac{m\omega }{\pi \mathrm{\hslash }}\right)}^\frac{1}{4}{H}_{n}\left(\sqrt{\frac{m\omega }{\mathrm{\hslash }}}x\right)\mathrm{exp}\left(-\frac{m\omega }{2\mathrm{\hslash }}{x}^{2}\right)$$

with the corresponding eigenenergy ${E}_{n}=n\mathrm{\hslash }\omega$, and ${H}_{n}$ is the $n$ th Hermite polynomial^43^.

Finally, we obtain the general solution of the FP equation whose time-independent solution is a mixed $\chi$ distribution by transforming the solution of the Schrödinger equation into that of the FP equation, which leads to the PDF in the following form

$$\rho \left(x\right)={\sum }_{n=0}^{\infty }\frac{{A}_{n}}{\sqrt{{2}^{n}n!}}\sqrt{\frac{m\omega }{\pi \mathrm{\hslash }}}\mathrm{exp}\left(-{E}_{n}\right)\times {H}_{n}\left(\sqrt{\frac{m\omega }{\mathrm{\hslash }}}x\right)\mathrm{exp}\left(-\frac{m\omega }{\mathrm{\hslash }}{x}^{2}\right)$$

where ${A}_{n}$ is an amplitude parameter determined by the initial distribution, which remains to be estimated. The random variable $x$ follows the Gaussian, Rayleigh, and Maxwell–Boltzmann distribution, etc. for $n=0, 1, 2, \cdots$. They all describe the displacement of a particle, that is, the first difference in log prices, in $(n+1)$ dimensional Euclidean space spanning Hilbert space.

## Results and discussion

### VR Test

Table 2 shows the VR test results for the daily log Bitcoin price series. The random walk hypothesis is partially rejected under the homoscedasticity assumption. Specifically, there is mixed evidence: the log of Bitcoin’s price series follows the EMH in the long-run ($\mathrm{VR}(q)\approx 1$) in line with previous literature^12,44,45^, while it appears to have a mean-reverting property ($\mathrm{VR}\left(q\right)<1$) in the short-run which is consistent with Corbet and Katsiampa^46^.

**Table 2:** VR test results on the log price series of Bitcoin. Variance ratios for holding periods 2, 4, 8, and 16 are tested under the homoscedasticity assumption. ** and *** indicate significance at the 1% and 0.1% levels, respectively.

	*q* = 2	*q* = 4	*q* = 8	*q* = 16
VR(*q*)	$0.721$***	$0.715$***	$0.798$**	$0.934$
(*p*-value)	($0.000$)	($0.000$)	($0.002$)	($0.491$)

To ensure the adequacy of the VR test, it must hold that the innovations of log price series, that is, the log return series, are Gaussian white noise. However, several previous studies have shown that power law emerges in the tail distribution of Bitcoin’s return series^47–50^. Accordingly, we conduct normality tests on the residuals of random walk specification. Table 3 indicates that the null hypothesis, *the innovations of the log price series are normally distributed*, is rejected at the 1% significance level for the two test statistics such as Jarque–Bera and Shapiro–Wilk tests regardless of sampling intervals. This indicates that the VR test is limited, requiring another approach.

**Table 3:** Normality test results on the log return distribution of Bitcoin. Jarque–Bera test is run by ${\chi }^{2}$ statistic, and Shapiro–Wilk test is by $W$ statistic. The cutoff values for $W$ are calculated through Monte-Carlo simulations. *** indicates significance at the 0.1% level. The sampling interval *q* is applied differently to the same data.

*H*_*0*_ ∶ The data follow a normal distribution			
Obs	*q*	Jarque–Bera	Shapiro–Wilk
2,133	1	$1.610\times {10}^{5}$***	$0.676$***
2,132	2	$2.927\times {10}^{4}$***	$0.786$***
2,130	4	$7.773\times {10}^{3}$***	$0.856$***
2,126	8	$3.812\times {10}^{3}$***	$0.882$***
2,118	16	$3.150\times {10}^{3}$***	$0.879$***

### QHO

**Figure 1:**
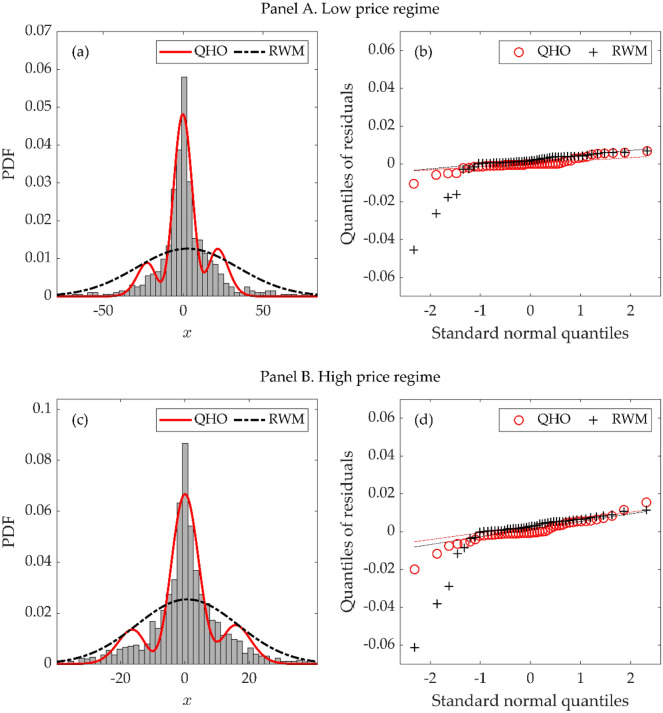
Model estimation results. Panels (**a**) and (**c**) plot the PDFs of each estimated model in the low and high price regimes, respectively. Panels (**b**) and (**d**) describe Q–Q plots for the residuals of each model by standardized normal order, plotted with a regression line, in both regimes. Both models are estimated using a daily sampling interval (*q* = 1). Outliers that deviate from the mean by more than $2.58\times \mathrm{Std}.\mathrm{ Err}.$ are excluded.

Before testing the market efficiency through the QHO, we investigate whether the QHO clearly explains the log return distribution of Bitcoin. Here, we divide the overall sample period into two sub-periods, i.e., low and high price regimes, as the dynamics of Bitcoin price appear differently due to the significant difference in its price level^51^. The low price regime extends from September 1, 2010, to February 26, 2013, and the high price regime spans from February 27, 2013, to March 31, 2019. Figures 1 (a) and (c) show the histograms of log returns in the Bitcoin market and the PDFs estimated from (i) the QHO and (ii) the random walk model (RWM) in the two regimes. Figures 1 (b) and (d) display the quantile–quantile (Q–Q) plots for the residuals of each model. The PDFs estimated by the QHO fit well, while those by the RWM significantly underestimate the data at the zone near zero, regardless of the price regimes. This is confirmed through two goodness of fit tests, i.e., Kolmogorov–Smirnov and Cramer-von Mises tests (Table 4). Moreover, the likelihood-ratio (LR) test compares the goodness of fit of two competing models based on the ratio of their likelihoods and further elaborates that the QHO describes the data better (Table 5).

**Table 4:** The goodness of fit test results on the log return distributions of Bitcoin derived from QHO and RWM. The Kolmogorov–Smirnov test is run by $D$ and Cramer-von Mises test is by $T$ statistics. The cutoff values for $T$ statistics are calculated through Monte-Carlo simulations. In the test, outliers that deviate from the mean by more than $2.58\times \mathrm{Std}.\mathrm{ Err}.$ are excluded. The numbers in parentheses are *p*-values of the statistics, where *** represents significance at the 0.1% level.

*H*_*0*_ ∶ Assumed model fits the distribution of data				
	QHO		RWM	
Period	*D*	*T*	*D*	*T*
Low price regime	0.038 (0.354)	0.186 (0.299)	0.207*** (0.000)	12.051*** (0.000)
High price regime	0.031 (0.110)	0.429 (0.061)	0.144*** (0.000)	15.182*** (0.000)

**Table 5:** LR test results. The LR test assesses the adequacy of the QHO model over RWM where outliers that deviate from the mean by more than $2.58\times \mathrm{Std}.\mathrm{ Err}.$ are excluded. The result indicates that the QHO (unrestricted) model explains the data better than the RWM (restricted) model. The formula of the LR test statistic is $LR=-2\mathrm{log}({L}_{r}/{L}_{u})$, where ${L}_{u}$ and ${L}_{r}$ denote the likelihood of the respective models such as QHO and RWM. The test statistic follows ${\chi }^{2}$ distribution with the degree of freedom (*d.f.*) 3, which refers to the number of constrained parameters. The *p*-value indicates that the unrestricted model outperforms the restricted model.

*H*_*0*_ ∶ There is no difference between both log-likelihood values					
Period	*d.f*	QHO $\left(\mathrm{log}{L}_{u}\right)$	RWM $\left(\mathrm{log}{L}_{r}\right)$	LR test stat. (${\chi }^{2}$)	*p*-value
Low price regime	3	–2640	–2707	133	0.000
High price regime		–5590	–5755	330	0.000

In Table 6, the mean squared error (MSE) and mean absolute error (MAE) show that the PDFs derived from the QHO (mixture $\chi$ distribution) have smaller errors than those from the RWM (Gaussian distribution) in both price regimes. This is because the solution of the QHO is a generalized solution of the RWM, capturing both Gaussian and non-Gaussian features together. Particularly, the QHO includes market uncertainty through the properties of superimposed wave functions and assumes that the resilience of the harmonic oscillator reflects the restoring force that drives market return to the long-run equilibrium^26^: market friction is reflected in the stochastic differential equation.

**Table 6:** Prediction errors of the log return distributions of Bitcoin derived from the QHO and RWM. Each estimated value is calculated based on the daily interval.

	QHO	RWM
*Panel A. Low price regime*		
MSE	$2.861\times {10}^{-6}$	$2.339\times {10}^{-5}$
MAE	$5.979\times {10}^{-4}$	$1.417\times {10}^{-3}$
*Panel B. High price regime*		
MSE	$1.024\times {10}^{-5}$	$4.935\times {10}^{-5}$
MAE	$1.250\times {10}^{-3}$	$2.173\times {10}^{-3}$

Table 7 summarizes the estimated probabilities assigned to the first three low-lying eigenstates ($$n=0, 1, 2)$$ for Bitcoin in both regimes as well as in the overall sample period (Those for the benchmarks in the overall sample period are shown in the supplementary information). Each eigenstate of the QHO model is described as a $$\chi$$ distribution, and the probability $${P}_{n}$$ assigned to each represents a value of the probability assigned to each $$\chi$$ distribution^26^. The state of $$n=3$$ and higher is neglected because the values assigned to $${P}_{n}$$ are less than $${10}^{-4}$$. The result is presented along with daily ($$q=1$$) and other different holding periods ($$q=2, 4, 8, 16$$). Particularly, since a market is efficient when the log return distribution is close to the Gaussian distribution, it can be said that the Bitcoin market follows the EMH based on the value of $${P}_{0}$$: that is, the probability allocation in the Gaussian distribution that describes the ground state of the QHO model. Unlike the mixed evidence of the VR test, the result of QHO consistently supports the efficiency of the Bitcoin market: the log of Bitcoin price follows a random walk with a probability of 90% or more.

**Table 7:** Probability assigned to each eigenstate in the Bitcoin market. The holding period ($q$) reflects the $q$ th difference of the log price data as ${x}_{t}=252.5/q \left(\mathrm{ln}{p}_{t}-\mathrm{ln}{p}_{t-q}\right).$

$q$	${P}_{0}$	${P}_{1}$	${P}_{2}$
*Panel A. Overall sample period*			
1	0.907	0.003	0.090
2	0.919	0.007	0.074
4	0.947	0.012	0.041
8	0.948	0.018	0.034
16	0.963	0.028	0.009
*Panel B. Low price regime*			
1	0.901	0.006	0.093
2	0.903	0.010	0.087
4	0.925	0.024	0.051
8	0.938	0.034	0.028
16	0.928	0.061	0.011
*Panel C. High price regime*			
1	0.908	0.003	0.089
2	0.924	0.006	0.070
4	0.948	0.009	0.043
8	0.938	0.010	0.052
16	0.981	0.013	0.006

### Discussion

Our results are explained through market capitalization and the speed of information discovery. Table 8 shows that the $m\omega$ value of Bitcoin is much smaller with the scale being about 1/19 to 1/47 than that of other assets (e.g., commodity, stock, and currency). According to Ahn et al.^26^, $m$ can be interpreted as the market capitalization and $\omega$ as the angular velocity for log return fluctuations. The average market capitalization ($m$) of Bitcoin in 2010–2019 is about 1/321 and 1/648 of the gold and S&P 500, respectively. Thus, the oscillating velocity around the equilibrium ($\omega$) for Bitcoin is estimated about 17 and 25 times larger than those for the gold and S&P 500, respectively. It implies that information circulation in the Bitcoin market is sufficiently fast compared to others, confirming the efficiency of the Bitcoin market together with the probability assigned to the ground state.

**Table 8:** Market capitalization and the speed of information discovery. The $m\omega$ values are estimated through the QHO model. Market capitalization represents the average daily market capitalization during the sample period. The daily market capitalization of Bitcoin and S&P 500 is retrieved from Quandl.com and the Center for Research in Security Prices, respectively. The daily market capitalization of gold is estimated by multiplying daily gold prices with the yearly above-ground gold reserves provided by World Gold Council. As the definition of market capitalization of USD/EUR is ambiguous, we have not directly made a comparison with the $\omega$ of USD/EUR. The period of data collection was from September 10, 2010, to March 31, 2019. To match the sample period of Bitcoin and that of the other assets, we extract daily data excluding the holidays for Bitcoin.

	Bitcoin	Gold	S&P 500	USD/EUR
$m\omega$	$9.067\times {10}^{-36}$	$1.685\times {10}^{-34}$	$2.367\times {10}^{-34}$	$4.297\times {10}^{-34}$
Market capitalization (USD)	$2.707\times {10}^{10}$	$8.679\times {10}^{12}$	$1.754\times {10}^{13}$	–

**Figure 2:**
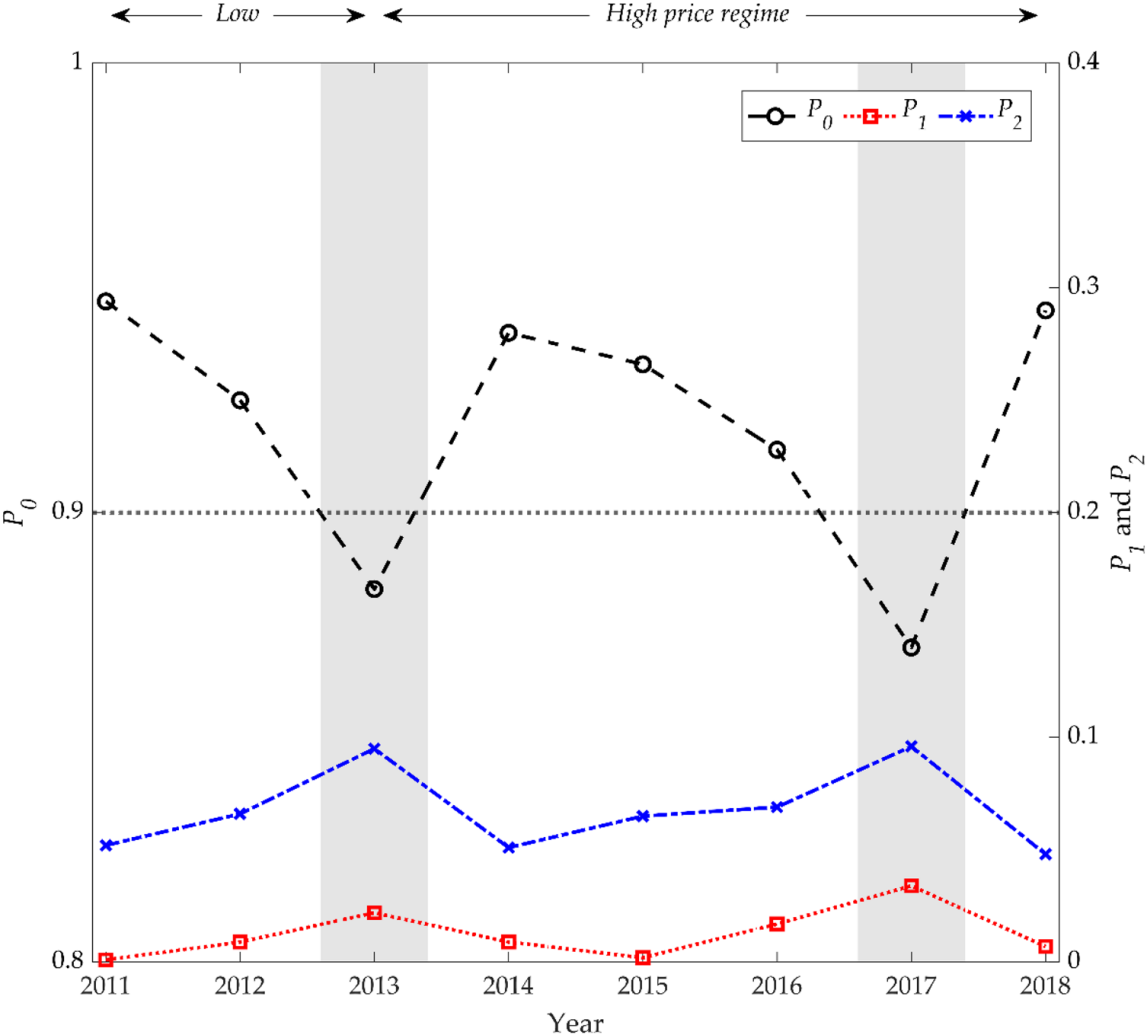
Yearly change of the probability allocation in each eigenstate in the Bitcoin market. The black dashed line shows the probability assigned to the ground state (${P}_{0}$) while the blue dash-single dotted line and red dotted line describe the probabilities assigned to the first (${P}_{1}$) and second excited state (${P}_{2}$), respectively. Two shaded areas indicate the periods when ${P}_{0}$ value is estimated below 0.9.

We estimate the probability assigned to the first three low-lying eigenstates for each year. Figure 2 shows that the yearly ${P}_{0}$ value fluctuates over the 90% level. This further confirms the robustness of our results: there is no significant change in the pattern of probability allocation at the ground state by year. This implies that technical analysis of historical price series cannot provide a long-term advantage in the Bitcoin market; future price evolution will be based on new information rather than past price performance. However, the ${P}_{0}$ values in 2013 and 2017 fell to around 88% and 87%, respectively. On the one hand, the decrease in 2013 is regarded as the transition period, shifting the Bitcoin market from a low to a high price regime^51^. The Bitcoin price, which had been in the tens of US dollars, exceeded USD 1,000 per BTC and burst a market bubble^52^: in December 2013, the Bitcoin price surged to USD 1,147 per BTC, then plunged by up to 85% in the following year. Moreover, in 2013, Bitcoin production (mined volume) drastically decreased due to increased mining difficulty. This significantly affected the price of Bitcoin on the supply side^53^. Put differently, during the transition period of the price regime, the strong collective phenomenon together with supply shortage resulted in a decline in the efficiency of the Bitcoin market; the ${P}_{0}$ value supports these arguments well.

However, in 2017, there was a large increase of public interest in cryptocurrencies, which caused higher levels of uncertainty and induced herding behavior in cryptocurrency markets^54^. Bouri et al.^55^ statistically confirmed this by showing that significant herding frequently occurred in 2017 in cryptocurrency markets. Accordingly, in line with Froot et al.^56^, the decreased ${P}_{0}$ value in 2017 can be explained as follows: many short-term speculators who are new to cryptocurrency herd in the Bitcoin market based on information completely irrelevant to fundamentals. Meanwhile, this increase in irrational speculation was mainly derived from China’s cryptocurrency exchange market which at that time accounted for more than 90% of the global cryptocurrency trading volume. Accordingly, the People’s Bank of China banned domestic cryptocurrency trading and initial coin offerings in September 2017, followed by a drastic cryptocurrency price drop and resurgence a few days after^57^. Nonetheless, after these strict regulations were imposed, most of the trading volume of cryptocurrencies in China moved elsewhere (e.g., U.S., Japan, and South Korea) and their impact on cryptocurrency prices was ambiguous^58^. This implies that such strict regulations in cryptocurrencies would not be effective but just increase price uncertainty, affecting their market efficiency, which can also explain the low ${P}_{0}$ value in 2017.

**Figure 3:**
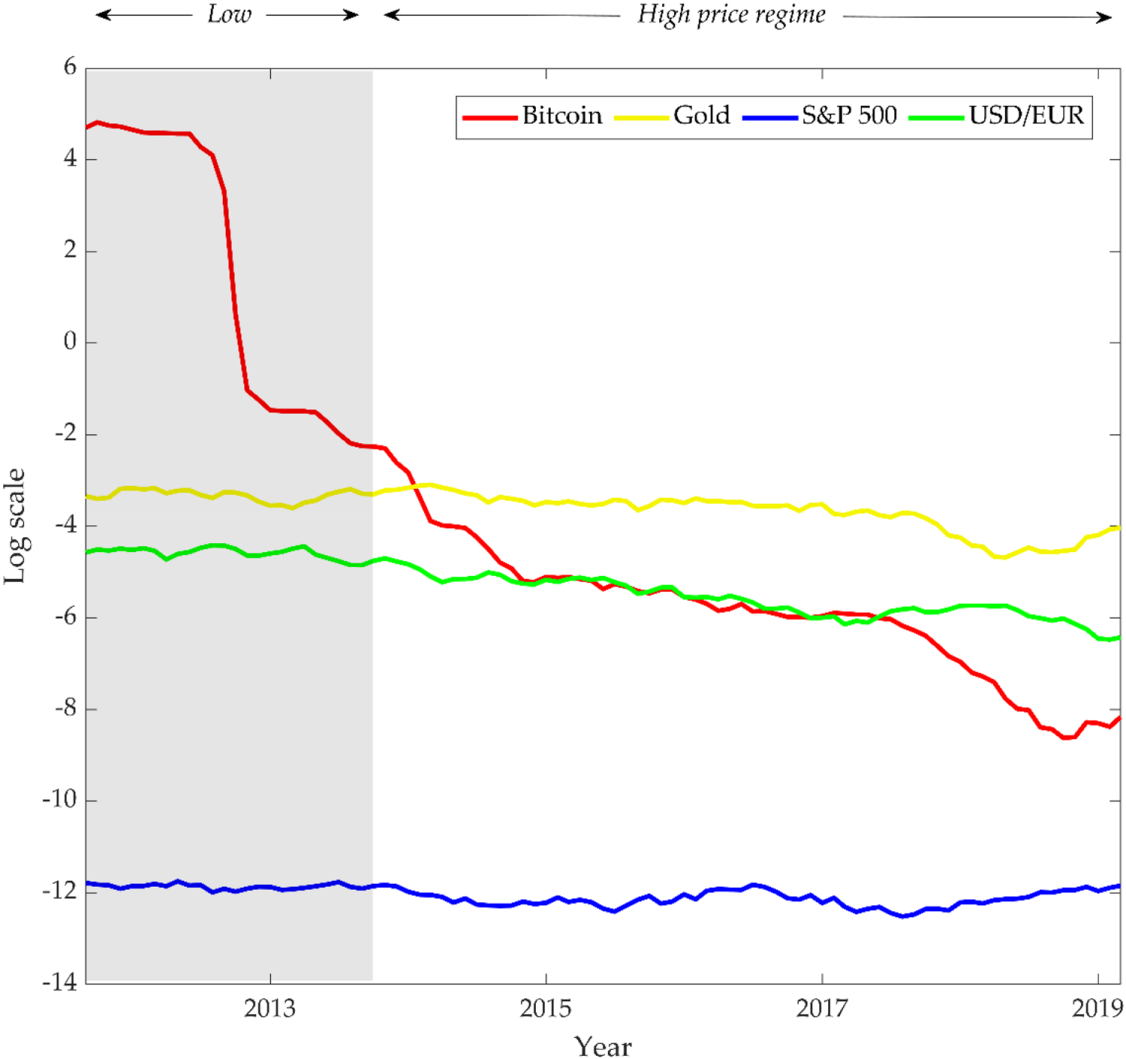
Amihud illiquidity of the Bitcoin, gold, S&P 500, and USD/EUR markets. Following Amihud ^63^, the monthly illiquidity is estimated using the daily history of trades. The smaller the calculated illiquidity value, the greater the liquidity of the market. The red, yellow, blue, and green lines indicate Amihud illiquidity of the Bitcoin, gold, S&P 500, and USD/EUR markets, respectively. Each colored line is expressed on a log scale for comparison purposes, using data available from September 2010 to March 2019. The shaded area indicates the period of the low-price regime. The trading volume for each asset were retrieved from the following websites: https://data.bitcoinity.org/markets (Bitcoin), https://backtestmarket.com (gold), https://finance.yahoo.com (S&P 500), and https://www.fxcm.com/markets (USD/EUR).

Liquidity increments in the Bitcoin market positively affect market efficiency; particularly, liquidity change plays a central role in the return predictability and, thus, informational efficiency of cryptocurrencies^16,18^. Generally, liquidity increase improves market efficiency through two channels^59,60^: first, it enables faster and cheaper transactions, thereby price promptly absorbs available information in the marketplace; second, it encourages investors to easily make tiny- or short-term massive transactions based on private information that barely impacts prices, approximating the market prices to fundamental asset value^61–63^. Therefore, we examine the Amihud illiquidity ratio^64^ and explain the market efficiency of Bitcoin with the changes in liquidity: Fig. 3 shows that the Bitcoin market’s liquidity keeps increasing gradually, even surpassing the USD/EUR and gold since 2014.

Unlike other assets, Bitcoin has a unique market structure where *inelastic* supply meets *elastic* demand since the total issuance (mining volume) is predetermined. Therefore, investors’ reaction to market information has a huge impact on Bitcoin prices. Additionally, year-round 24/7 trading, relatively low transaction costs, and low entry barriers to the market help investors participate freely and make decisions promptly, ultimately facilitating quick information dissemination in the market price. This explains how the Bitcoin market was able to operate close to an efficient market even with a low liquidity level in the early stage, especially during the low price regime in 2010–2012. However, since 2013, the market has stabilized with the birth of multiple exchanges and institutional investors’ interests. Accordingly, the liquidity of the Bitcoin market continued to expand, which has further contributed to market efficiency in the high price regime. Thus, negative factors that inhibit the efficient dissemination of information in the market, such as instability of the initial market system, early government regulations, externalities like hacking, and highly speculative transactions, appear to be offset by the development of the Bitcoin market.

## Conclusions

This study examines the market efficiency of Bitcoin. As a first step, the VR test, which is widely used in literature, shows mixed evidence of the Bitcoin market efficiency. However, we confirm that the VR test is not suitable for testing the EMH because its basic assumption is not satisfied in the Bitcoin market: the increments of log price series are not Gaussian white noise. Consequently, we applied the QHO, which provides a general solution for the PDF of the log return. The probability assigned to the ground state indicates that the Bitcoin market is rather close to the efficient market, similar to other asset markets. We explain that the continual increase in the market’s liquidity and the change in price regime (from low to high) contribute to our findings. Additionally, the year-round 24-h trading system contributed to ensuring that market information is well reflected in the market price.

Our results show that the Bitcoin market is close to being weakly efficient, implying that developing a profitable trading strategy simply based on past trends of Bitcoin price is difficult for speculators. Moreover, considering the liquidity and market capitalization, Bitcoin and major altcoins are getting closer to other assets and thus, have a direct/indirect impact on the real economy both now and in the future. Therefore, policymakers should closely monitor Bitcoin’s market efficiency to avoid market failure when implementing policies and regulations that could affect its market size and liquidity or induce investors’ herding behavior.

## Supplementary Information

Supplementary Information.

## Data Availability

The datasets used and/or analyzed during the current study are available from the corresponding author upon reasonable request.
