# Supplementary material for: Market efficiency of cryptocurrency: evidence from the Bitcoin market
Source: Sci Rep. 2023 Mar 23;13:4789. doi: 10.1038/s41598-023-31618-4 (PMC10036534; doi:10.1038/s41598-023-31618-4)
Supplement: Supplementary file 1 — Supplementary Information. [file 41598_2023_31618_MOESM1_ESM.docx]

**Appendix**

In Table A1, we show probabilities assigned to the first three low-lying eigenstates ($n=0,1,2$) for gold, S&P 500, and USD/EUR. They have $P_{0}$ values larger than 0.9 and even closer to unity, implying that the gold, S&P 500, and USD/EUR markets follow the *weak-form* EMH.

|  | $P_{0}$ | $P_{1}$ | $P_{2}$ |
| --- | --- | --- | --- |
| Gold | 0.979 | 0.000 | 0.021 |
| S&P 500 | 0.956 | 0.005 | 0.039 |
| USD/EUR | 0.986 | 0.000 | 0.014 |

**Table A1.** The probability assigned to the first three low-lying eigenstates of gold, S&P 500, and USD/EUR.
